# Connect To Care (C2C): protocol for two-site randomized controlled pilot trial to improve outcomes for patients with hazardous drinking and PTSD and/or depression symptoms

**DOI:** 10.1186/s13722-023-00403-z

**Published:** 2023-08-17

**Authors:** Michael A. Cucciare, Kathy Marchant, Cristy Benton, Deanna Hildebrand, Sharfun Ghaus, Xiaotong Han, Ronald G. Thompson, Christine Timko

**Affiliations:** 1 Center for Mental Healthcare and Outcomes Research, Central Arkansas Veterans Affairs Healthcare System, North Little Rock, AR 72205 USA; 2 Education and Clinical Center, Veterans Affairs South Central Mental Illness Research, Central Arkansas Veterans Healthcare System, North Little Rock, AR 72205 USA; 3 Department of Psychiatry, University of Arkansas for Medical Sciences, Little Rock, AR 72205 USA; 4 Center for Innovation to Implementation, Department of Veterans Affairs Health Care System, Palo Alto, CA 94304 USA; 5 Department of Psychiatry and Behavioral Sciences, Stanford University School of Medicine, Stanford, CA 94305 USA; 6 Center for Innovation to Implementation, 795 Willow Road (152-MPD), Menlo Park, CA 94025 USA

**Keywords:** Alcohol, Strengths-based case management, Care initiation, Care engagement, Primary care, PTSD, Depression, Shared decision-making

## Abstract

**Background:**

In studies of the general population and of military veterans, many primary care patients with hazardous drinking and PTSD and/or depression (abbreviated here as HD +) do not initiate or engage with alcohol-related care. To address this gap in care, we identified and will pilot test a promising evidence-based intervention, Connect To Care (C2C). C2C is a strengths-based approach, delivered by a Care Coach by telephone and/or video, with four components: (1) identifying and leveraging patient strengths to facilitate care initiation, (2) collaborative decision-making around a menu of care options, (3) identifying and resolving barriers to care, and (4) monitoring and facilitating progress toward care initiation by, for example, checking on barriers, identifying solutions, and revisiting care options.

**Methods/Design:**

Aim 1 will involve adapting C2C for use in Veterans Affairs’ (VA) primary care. We will use an iterative process that includes focus groups and semi-structured interviews with key stakeholders (patients, primary care providers, and VA national policy leaders). In Aim 2, we will conduct a two-site, pilot randomized controlled trial to determine the feasibility of conducting a larger scale trial to test C2C’s effectiveness, ascertain the acceptability of C2C among primary care patients with HD + , and explore the efficacy of C2C to improve veteran patients’ initiation of and engagement in alcohol care, and their alcohol and mental health (PTSD, depression) outcomes, at 3-month follow-up. We will explore explanatory mechanisms by which C2C is effective.

**Discussion:**

Study findings are likely to have implications for clinical practice to enhance current approaches to linking patients with HD + to alcohol care by applying a practical intervention such as C2C. The results may improve treatment outcomes for people with HD + by drawing on patients’ strengths to problem-solve barriers to care following a process of shared decision-making with a coach. In addition to possibly accelerating the translation of C2C into practice, study findings will also support additional research in terms of a planned effectiveness-implementation hybrid trial, adding to this study’s potential for high impact.

*Trial registration*: ClinicalTrials.gov Identifier: NCT05023317.

## Introduction

Hazardous alcohol use (drinking more than recommended limits, including a screening score indicating probable alcohol use disorder [AUD] [1]) occurs at high rates among primary care patients. In the US, up to 20% of adult primary care patients engage in hazardous drinking (HD) [2], and the rate among US military veterans (henceforth referred to as “veterans”) seeking primary care may be as high as 30% [3–5]. HD is associated with high rates of co-occurring PTSD and depression, especially among veterans. A meta-analysis of epidemiological surveys found that people with AUD had 2.4 times greater risk of having major depression [6], whereas veterans with an AUD were four times more likely to meet diagnostic criteria for depression or PTSD as those without an AUD [7]. A systematic review found that in most of the 42 studies included, the prevalence of PTSD among persons engaged in HD was > 10% [8]. However, among veterans, co-occurring PTSD and depression were found among 25% and 38%, respectively, of those engaged in HD [9]. Veterans who engage in HD and have PTSD or depression have poorer alcohol treatment outcomes; more anger, marital, and legal problems; and greater risk of poor quality of life, suicide attempts, and death than veterans without these comorbidities [10–13].

The high co-occurrence of HD with PTSD and depression is concerning because alcohol use worsens symptoms of these conditions [14, 15]. However, treating HD helps to alleviate PTSD and depression symptoms [16, 17]. Even so, many primary care patients with HD, PTSD, and/or depression, both civilians and veterans, do not initiate or engage with alcohol-related care [18, 19]. Indeed, national US data indicate that persons with AUDs commonly utilize health care and are often screened about alcohol use, but few receive alcohol treatment, supporting the view that health care settings, such as primary care, represent an important opportunity to introduce alcohol treatment to improve patient outcomes [20]. More specifically, among 1,172,606 positive screens documenting HD, representing 830,825 veteran patients, only 127,259 (10.9%) received specialty addiction treatment within one year [18]. However, the same study found that 35.9% (n = 297,924) of patients with positive screens met VA clinical guideline criteria for needing treatment (i.e., AUDIT-C score ≥ 8) [18]. Together, these findings highlight the importance of linking patients with HD and PTSD and/or depression (henceforth referred to as HD +) to alcohol care to improve the outcomes of care initiation and engagement, alcohol use, and mental health symptoms.

To that end, we identified and will pilot test a promising evidence-based intervention, Connect To Care (C2C). C2C is an evidence-based practice that leverages the telephone and video health care delivery infrastructure augmented during the COVID pandemic, and has not yet been evaluated with veterans*.* It is a strengths-based approach that includes four components delivered by a Care Coach. In this context, a Care Coach is someone with experience providing care to persons in treatment for AUD such as a master’s level social worker or mental health counselor, nurse, or peer recovery specialist. The four components of C2C are: (1) identifying and leveraging patient strengths to facilitate care initiation, (2) collaborative decision-making around a menu of care options, (3) identifying and resolving barriers to care, and (4) monitoring and facilitating progress toward care initiation by, for example, checking on barriers, identifying solutions, and revisiting care options. Strengths-based linkage interventions such as C2C have been effective in linking individuals with substance use disorders, some with co-occurring mental health symptoms, and persons with chronic health conditions, to care [21, 22]. Two meta-analyses studying patients using substances found strengths-based linkage interventions were associated with reduced need for inpatient services, greater treatment retention, improved quality of life, and greater satisfaction with the care process [23, 24]. Strengths-based linkage interventions are considered evidence-based practices by the Substance Abuse and Mental Health Services Administration [25] and Centers for Disease Control and Prevention [26].

**Fig. 1:**
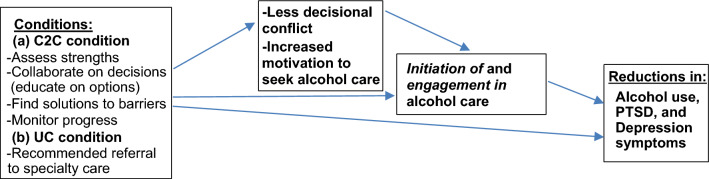
Conceptual model of Connect To Care (C2C) mechanisms

A strength-based approach such as C2C should help overcome key obstacles to patients initiating alcohol care, which include low motivation to seek treatment and uncertainty about which course of action to take (decisional conflict; Fig. 1). C2C can also increase engagement in alcohol care, leading to improved outcomes [27]. C2C aims to increase motivation by identifying and using patient strengths (Component 1) and educating patients about available evidence-based alcohol care options (pharmacotherapy, behavioral interventions, mutual-help, and ehealth (Component 2), which can reduce decisional conflict (Component 2). Providers educate patients about available care options using a menu describing the details of each option, including their pros and cons. This discussion focuses on deciding which care option is best for patients (including no care at this time if preferred by the patient) and helping to identify and resolve barriers (e.g., misconceptions about options, lack of transportation) to alcohol care (Component 3). To help reduce decisional conflict and increase motivation, C2C also uses the spirit of motivational interviewing to help patients decide whether to initiate care [28]. Patients are offered up to five telephone- or video-delivered meetings of C2C over seven weeks, during which progress is monitored (Component 4) to improve motivation and increase decisional certainty by reinforcing identified strengths that promote care initiation and offering additional education about options when needed. For some patients, receiving at least three sessions, compared to fewer than three, may be sufficient in that it results in a higher likelihood of initiating care [21].

When introducing the present study, it is important to consider how approaches like C2C differ from other approaches to connect primary care patients with HD + to alcohol care. Although other interventions such as Choosing Healthier Drinking Options in Primary Care (CHOICE) [29] and Substance Use Motivation and Medication Integrated Treatment (SUMMIT) [30] are more effective than usual care for linking patients with HD from primary care to alcohol care, their linkage rates are low. CHOICE, which combines medication for AUD, collaborative care, and brief behavioral intervention for patients with HD or HD + , had more receipt of AUD medication (32%) than Usual Care (UC) (8%) at 12-month follow-up [50]. No intervention effect was observed on linkage to other alcohol care options (e.g., addiction treatment, mutual-help groups) or drinking outcomes. SUMMIT is a collaborative care intervention offering brief therapy in primary care or referral to a primary care provider for medications for AUD or opioid use disorder. SUMMIT participants (some with depression) were more likely than usual care participants to see a primary care therapist to discuss alcohol use (36% vs. 11%) and to be abstinent at 6-month follow-up (33% vs. 22%) but there was no difference in medication use (13% in each arm) [30]. An important aspect of CHOICE and SUMMIT is their emphasis on AUD medication since some patients with HD or HD + may not need or want medication for AUD [31]. In contrast, C2C does not emphasize any single alcohol care option; indeed, it offers a menu of care options. Also, C2C engages participants who are not interested in changing their alcohol use at the present time in continued decision-making rather than repeatedly administering brief interventions (CHOICE) or encouraging an initial therapy appointment (SUMMIT).

We hypothesize that primary care patients with HD + will benefit from a strengths-based approach in terms of initiating and engaging in needed care and achieving better alcohol and mental health outcomes. However, C2C has not been studied in the Veterans Health Administration (VA) system or with veterans with HD + , and has not been evaluated with delivery by telephone or video, which is needed given changes in the delivery of health care during the pandemic. For this reason, adapting C2C for this veteran population in the primary care setting (Aim 1), and conducting a pilot study to obtain preliminary evidence for C2C’s acceptability, feasibility, and efficacy (Aim 2) are high priorities*.* Findings from this study will inform ongoing clinical practice, and be used in a subsequent study using a hybrid effectiveness-implementation design to examine both effectiveness and implementation outcomes [32].

## Methods

### Overview

Aim 1 of this study will adapt C2C for use in VA primary care. We will use an iterative process that includes focus groups and semi-structured interviews with key stakeholders (veteran patients, primary care providers [physicians, social workers, and psychologists], and VA national policy leaders). In Aim 2, we will conduct a two-site, pilot randomized controlled trial to determine the feasibility of conducting a larger scale trial to test C2C’s effectiveness, determine the acceptability of C2C among primary care patients with HD + , and explore the efficacy of C2C to improve veterans’ initiation of and engagement in alcohol care, and their alcohol and mental health (PTSD, depression) outcomes, at 3-month follow-up.

### Aim 1: adapt C2C for VA primary care

#### Procedure

To achieve Aim 1, we will use an iterative method, ADDIE: Analysis, Design, Development, Implementation, and Evaluation [33], that is commonly used in the field of instructional design to develop educational content. Analysis refers to sharing with key stakeholders the intervention’s goals (improving initiation of and engagement with alcohol care to benefit outcomes) and identifying stakeholder needs within the context of the target setting (primary care) and population (patients with HD +). Design refers to adapting the prototype of the C2C protocol based on information gathered during the Analysis phase. Development is the process of producing a final version of C2C (to be tested in Aim 2) based on feedback from stakeholders on the prototype. Implementation refers to putting C2C into action, i.e., implementing the adapted version in the target setting, including training the Care Coach to deliver C2C in Aim 2. The Evaluation phase consists of a formative evaluation to obtain feedback from stakeholders on C2C to optimize its acceptability for use in Aim 2.

We will recruit 12 veterans who screen positive for HD + in primary care, 6–10 providers (primary care physicians and providers in integrated primary care-mental health), and 6–10 national policy leaders to participate in semi-structured interviews. Half of the veterans and providers will be recruited from each of the two study sites. Sample sizes were determined using recommendations for the number of interviews needed to obtain theoretical saturation, which is the point at which no new insights emerge from additional interviews [34, 35]. National policy leaders will include VHA experts in primary care medicine and in the treatment of mental health and substance use disorders.

#### Recruitment: patients

To recruit veterans, we will use the VA Corporate Data Warehouse (CDW; a national level database housing clinical, administrative, and financial information) to identify potentially eligible veterans seen in primary care at each of the two sites. We will identify veteran patients with HD + : (1) an AUDIT-C score of ≥ 5 (used by VA to indicate HD) and (2) a positive screen for PTSD and/or depression, i.e., Primary Care PTSD Screen for DSM-V (PC-PTSD-5) score of ≥ 3 [36] and/or Patient Health Questionnaire-2 (PHQ-2) score of ≥ 3 [37] in a VA primary care visit within the past year. We will invite identified patients to participate in the study.

Specifically, we will randomly select a subsample of patients from the CDW dataset to mail opt-out invitation letters. We expect women will represent about 3–10% of identified patients. Potential participants will receive a study packet that includes an invitation letter, an informed consent document, and notification that we will contact them by telephone (should they not opt-out) 10 days following the mailing. The study will be presented to potential participants as research to learn more about VA and community resources to improve their health. Study staff will explain to potential participants that during a recent health care visit they answered questions about their alcohol use in a way that indicated possible difficulties around drinking. Research assistants will confirm eligibility of interested patients by re-administering screens to determine whether patients continue to meet screening criteria for HD + . Research assistants will also assess cognitive functioning using the Montreal Cognitive Assessment (MOCA) section on orientation [38]. Veterans unable to answer orientation items with reasonable accuracy and whose interview suggests likely cognitive impairment will be ineligible. Research assistants will answer questions about study participation and obtain informed consent from interested and eligible patients. Participants will be compensated for each in-person interview.

#### Recruitment: providers

To recruit providers, at each site, project staff will email providers to briefly explain the project’s purpose and invite them to contact the study team to complete an interview. Emails will be followed with a phone call to answer questions, obtain informed consent, and schedule interviews (in person or by phone, while on or off duty, as providers prefer). We will use similar procedures to recruit national policy leaders to complete phone interviews.

#### Interviews: data collection

In the interviews, participants will be asked to review a handout, elaborated upon by the interviewer, providing a description of C2C. Interviewees will be asked about the core C2C components, such as which parts may be hard to understand, how to best inform patients with HD + about care options, and duration of the intervention (e.g., whether C2C provides enough monitoring). Interviews will help ensure that C2C components (e.g., length of time between sessions, how patients who do not want help are approached) are appropriate for use with veterans with HD + and for use within VA. Implementation-related questions will assess the feasibility of using well-established procedures (e.g., training, facilitation) for rolling out evidence-based interventions throughout VA [39]. Feedback obtained will inform efforts to implement C2C in VA primary care in a subsequent, fully powered hybrid trial. All interviews will be audio recorded and transcribed to facilitate data analysis.

#### Interviews: data analysis

Data collected from the interviews will be analyzed using a rapid analytic technique called template analysis, a method of team-based qualitative analysis, which organizes and summarizes data into predefined conceptual domains [40, 41]. Project staff will read all interview transcripts to identify participants’ opinions about C2C and obtain suggested modifications for adapting C2C to the target population and clinical setting. Prototype templates will be created in electronic documents containing broad conceptual domains (e.g., modifications to the content and/or structure of each C2C session, training needs of providers to learn C2C). After developing protocol templates, project staff will combine individual templates into a summary template, with content grouped into categories within each conceptual domain. To establish analytic validity, template content will be illustrated with verbatim quotations from participants, which will provide verification of the accuracy of content labelling and grouping. Results will be used to develop a final version of C2C for use in Aim 2.

### Aim 2: examine C2C’s feasibility, acceptability, and efficacy in two-site pilot trial

#### Eligibility

To be eligible for Aim 2, veterans (n = 140) will (1) have screened positive for HD + in the prior 12 months and have positive rescreens, (2) not have received ≥ 3 sessions of specialty substance use disorder treatment or participated in ≥ 2 weekly mutual-help groups in the past 30 days, (3) not have significant cognitive impairment, (4) have ongoing access to a mobile or landline telephone, (5) provide at least one contact who will know their contact information, and (6) not have participated in an interview for Aim 1.

#### Recruitment

For a fully powered randomized controlled trial, we will need to recruit 360 Veterans (adjusting for an expected 75% retention rate at 6-month follow-up). Specifically, considering a medium effect size of C2C, a sample size of 360 will be needed to achieve 80% power to detect a treatment effect between groups (on care initiation) with a type 1 error rate of 0.05. To achieve a sample size of 360, we will need to recruit 10 veteran patients per month over a 36-month period. Therefore, in the planned pilot study, we will determine the feasibility of recruiting 10 Veterans per month (5 at each site, a rate of about one participant per week per site) over a 14-month period.

#### Procedure

**Fig. 2:**
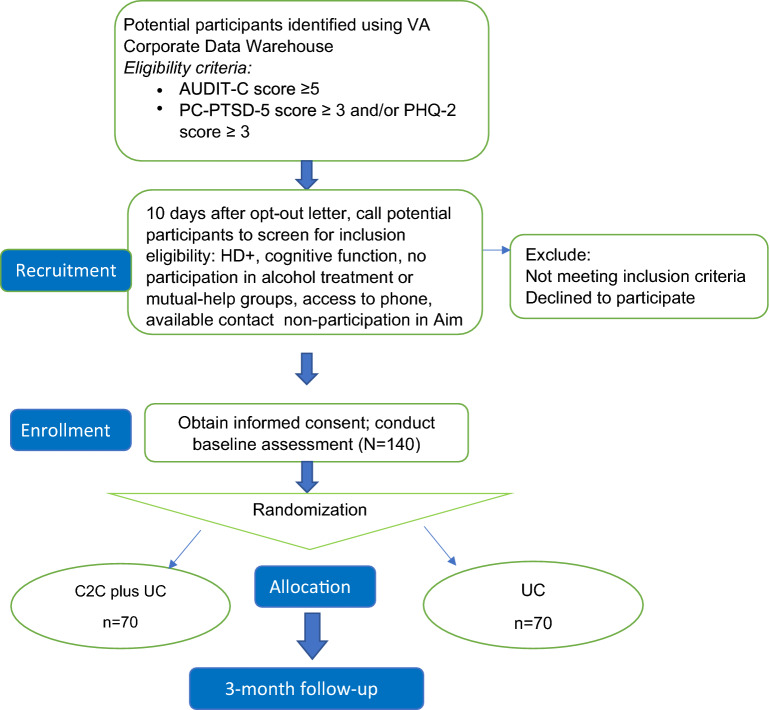
Flowchart of participants through the trial

Recruitment procedures for Aim 2 will follow procedures described for patients in Aim 1. Research assistants will confirm eligibility of interested patients by rescreening, using the same screeners, for HD + (to determine if they continue to meet study eligibility criteria) and determining eligibility on criteria that could not be prescreened with CDW data (cognitive function, have not participated in ≥ 3 alcohol treatment and ≥ 2 weekly mutual-help group sessions in the past 30 days, access to phone, available contact, non-participation in Aim 1). Patients who consent to participate will be randomly assigned to the UC or C2C condition using the Research Randomizer website after their baseline assessment. The flow of participants through the pilot trial is shown in Fig. 2.

#### UC condition

All participants will be eligible to receive UC delivered by their VA primary care physician that includes mandated brief alcohol counseling and referral to specialty substance use disorder care according to VA practice guidelines.

#### C2C condition

Veterans assigned to the C2C condition will receive UC plus the C2C intervention. They will be contacted by the study Care Coach by phone to schedule their first session. C2C will provide up to five telephone or video conference sessions over seven weeks. Sessions will last about 30–60 min, depending on the participant’s specific needs.

#### Care coach training and supervision

The same Care Coach will deliver C2C by telephone or video conference to participants at both study sites. The coach will participate in a two-day training on the C2C protocol and receive ongoing supervision. Training will begin with the rationale for the study, a review of the unique needs and challenges of veterans with HD + , and a summary of the literature on the effectiveness of strengths-based interventions, followed by observation and role-play exercises for delivering C2C and using the menu of alcohol care options. Specifics of the phone sessions will be reviewed: overall structure of each session; building rapport; how to discuss and assess personal strengths; identify whether the patient is interested in pursuing alcohol care; how to proceed if patients are not interested (continue encouraging, problem solving, monitoring) in care; how to proceed if patients are interested (present the menu of care options; help the patient choose an alcohol care option and support initiation); identify barriers to help-seeking and problem-solve solutions in a collaborative manner; and monitor progress. The coach will role-play phone and video conference sessions that will be audiotaped, coded for fidelity, and reviewed with supervision and feedback. After training, we will ensure the coach’s readiness to deliver C2C with a start-up practice phase. When the clinical supervisor agrees that the coach is consistently following the C2C protocol, the coach will be certified to begin conducting sessions with non-practice participants.

#### C2C fidelity

To assess the fidelity of the C2C intervention, the clinical supervisor will complete the C2C Fidelity Assessment [26], consisting of a checklist of core C2C components, after the first 10 phone calls (and 4 randomly chosen phone calls per month thereafter) between the Care Coach and participants. The C2C Fidelity Assessment will be modified to include content modifications arising from Aim 1. The coach will audio-record all sessions (with appropriate consent) so selected sessions can be evaluated for fidelity. Corrective feedback will be provided to the coach if needed. In addition, the coach will document any barriers or facilitators to intervention delivery that arise throughout the study.

#### Measures

*Feasibility* will be measured as: (a) the proportion of veteran patients screening eligible for the study who enroll, (b) monthly enrollment rates compared to the benchmark of at least 10 patients per month, (c) participant 3-month follow-up rates compared to a benchmark of $\ge$ 75%; and (d) C2C fidelity using the C2C Fidelity Assessment, i.e., a rating of $\ge$ 80% completion of the basic core elements of C2C [42]. *Acceptability* will be operationalized as (a) > 50% of patients randomized to C2C complete at least three sessions [21] and (b) score of > 18 on patients’ satisfaction with C2C, assessed with the VA Multisite Study’s measure of satisfaction with substance use disorder treatment [43].

To explore C2C’s *efficacy*, we will collect the following data at baseline and 3-month follow-up (exceptions are noted).

- Demographics: Age, sex, gender identity, race/ethnicity (baseline only), marital status, education, employment, income, and housing.
- Alcohol consumption: 90-day Time Line Follow-Back (TLFB) [44]. The TLFB is a calendar-based, retrospective self-report measure that assesses quantity/frequency of alcohol consumed. It is reliable and accurate when administered over the telephone [44]. Alcohol quantity and frequency will be measured using days and percent of heavy drinking days (≥ 4 standard drinks per day for women; ≥ 5 for men). In addition, AUD diagnosis and severity will be measured using the 11 items in the DSM-5 [45, 46].
- Depression: Patient Health Questionnaire-9 (PHQ-9) [47]. The PHQ-9 consists of nine questions that ask respondents how often they have “been bothered by any of the following problems” (with, e.g., sleep, energy, appetite) in the past two weeks (not at all = 0; nearly every day = 3) and are summed. Scores range from 0–27. Higher scores indicate more symptoms of depression. The PHQ-9 closely mirrors DSM criteria for depression and has excellent internal consistency and test–retest reliability.
- PTSD: PTSD Checklist-5 (PCL-5) [48]. The PCL-5 is a 20-item self-report measure that assesses the 20 DSM-5 symptoms of PTSD. A total symptom severity score (range = 0–80) is obtained by summing the score for each item. Higher scores indicate more severe PTSD symptoms.
- Drug use: Alcohol, Smoking and Substance Involvement Screening Test (ASSIST) [49]. The ASSIST will assess lifetime (baseline only) and past 90-day drug use, i.e., use of illicit drugs (stimulants, inhalants, street opioids) and non-medical use of prescription drugs (opioids, sedatives).
- Substance use and mental health care utilization (baseline only): Adapted version of the Addiction Severity Index (ASI) [50]. The ASI will be adapted to assess lifetime alcohol, drug, and mental health care utilization (VA and non-VA; baseline only).
- Substance use and mental health care utilization (follow-up only): Adapted TLFB. The TLFB method will be adapted to determine the primary efficacy outcomes of initiation of and engagement in alcohol care. Initiation of alcohol care is a dichotomous variable (yes/no) with “yes” defined as the participant reporting at least one of the following: attended an initial meeting with an outpatient or residential program; attended a mutual-help group meeting; received > 30-days’ supply of AUD medication; or accessed ehealth for alcohol use. Engagement in alcohol care will be defined using two indices: the percentage of days in the past 90 that participants obtained any alcohol care; and the total number of alcohol care options participants obtained over the past 90 days (scores will range from 0 = no care to 4 = obtained all types of care: outpatient/residential, mutual-help, medication, e-health). CDW data will also identify use of VA substance use and mental health care including initiation of treatment, type of care and setting, and frequency and duration of each care type.
- Mediators (see Fig. 1): Decisional conflict and motivation. Decisional conflict will be assessed with the Decisional Conflict Scale, a self-report measure used to assess decision uncertainty and discomfort [51, 52]. The scale’s 16 items yield five subscales (Informed, Values Clarity, Support, Uncertainty, Effective Decision) and a total score. The Readiness Ruler will assess motivation to initiate care [53]. It asks participants about their motivation to initiate alcohol care on a scale of 1 (less – not ready) to 10 (more – trying to seek care).

#### Assessment procedures

Research assistants, blinded to condition, will collect baseline and follow-up data from participants over the telephone. Each assessment will take about one hour. We will use an intent-to-treat design and follow participants who do not complete the intervention. Participants will be compensated for completing the assessments. To ensure high follow-up rates we will use proactive retention strategies, e.g., interviewers are comfortable with the study population and trained to establish rapport; toll-free telephone numbers for participants to report contact information changes. We will use these procedures to target a follow-up rate of at least 75%.

#### Analysis plan

We will examine the *efficacy* of C2C by comparing the two conditions (UC, C2C) on the primary outcomes of initiation of and engagement in alcohol care, and secondary outcomes of alcohol use, depression, and PTSD symptoms. To explore alcohol care initiation we will employ generalized estimating equations (GEE) or general linear models (GLM) using binomial distribution with logit link to determine whether participants differ by condition on alcohol care initiation at 3-month follow-up. To explore engagement in alcohol care we will use GEE or GLM models with cumulative logit or generalized logit to determine whether participants in the C2C condition had more engagement in alcohol care relative to UC over time. We will include a binary variable for study condition (C2C vs UC) and time for each assessment time point in the regression models and an interaction term between these two variables. Analyses for care engagement (percentage of days that alcohol care was obtained), alcohol (percent of drinking days and heavy drinking days), PTSD, and depression outcomes will be performed using GEE or GLM models to examine the effect of C2C over time. A normal distribution will be specified if an outcome is normally distributed; otherwise, an appropriate distribution (e.g., beta distribution for alcohol outcomes with percentages; gamma distribution for PTSD and depression outcomes) will be identified with the corresponding link specified. Variables for condition, time, site, and covariates will be included in all models along with the interaction term between condition and time. Models will be developed for each outcome separately.

We will also explore *mechanisms* (reasons C2C is effective*)* although the trial is not fully powered for this purpose*.* That is, decisional conflict and motivation to initiate alcohol care will be explored as potential mediators between condition and outcomes. We will use structural equation modeling (controlling for covariates) that corresponds to a hypothesized causal sequence among (1) C2C, (2) less decisional conflict and more motivation to seek alcohol care, (3) initiation of and engagement in alcohol care, and (4) alcohol, PTSD, and depression outcomes (Fig. 1). We will include the following paths simultaneously in the model: (1) condition (C2C vs UC) to decisional conflict and motivation to seek alcohol care, (2) decisional conflict and motivation to seek alcohol care to initiation of/engagement in alcohol care, and (3) initiation of/engagement in alcohol care to alcohol, PTSD, and depression outcomes. The dummy variables representing C2C and study site will be treated as exogenous; all other variables in the paths will be treated as endogenous. Other variables that are identified as strongly associated with condition at baseline may be included as exogenous. Model fit indices will be evaluated (e.g., RMSEA, SRMSR, CFI).

We will explore baseline levels of alcohol (probable AUD [AUDIT-C score > 8)] or not); PTSD (probable PTSD [PCL-5 score > 31] or not), and depression (moderate depression [PHQ-9 score > 10] or not) symptom severity as moderators of condition-outcome associations. We expect that all groups that receive C2C will benefit from it, but these analyses will provide data on whether C2C is especially helpful for patients with more or less severe baseline HD and PTSD and/or depression symptoms.

#### Debriefing interviews

At 3-month follow-up, we will conduct in-person debriefing interviews with 20 participants assigned to the C2C condition to obtain data on its feasibility, acceptability, and perceived efficacy (including reasons C2C is or is not helpful). Interviews will be audiotaped and transcribed to facilitate analysis. Participants will be compensated for completion of the interview. Patients who receive < 3 C2C sessions (n = 14; 7 at each site) and patients who receive $\ge$ 3 sessions (n = 6; 3 at each site) will be interviewed at the completion of their 3-month follow-up. In both subgroups, we will ensure that patients with probable AUD, probable PTSD, and at least moderate depression will be represented. Interviews will identify aspects of C2C that participants found helpful or not for initiating alcohol care and potential suggestions for improvement. Findings may inform strategies for deterring C2C dropout and improving C2C use and completion, as well as conceptual model development (e.g., identification of other potential mechanisms) of C2C.

Data collected from debriefing interviews will be analyzed using template analysis. Project members will read all interview transcripts to identify participants’ experiences participating in the C2C condition. Prototype templates will be created in electronic documents containing broad conceptual domains (e.g., what was your experience, what was helpful or not, what did you like and dislike) related to participating in C2C. Team members will combine prototype templates into a summary template (after reaching consensus on conceptual domains, aiming for 100% agreement) with content grouped into categories within each conceptual domain. The process of categorization will allow for the identification of experiences described by participants. To establish analytic validity, team members will illustrate template content with verbatim quotations from participants, which will provide verification of the accuracy of content labelling and grouping.

## Discussion

The aims of the current study are to adapt and examine an innovative, evidence-based approach to improving alcohol care initiation and engagement and improve health outcomes among veteran primary care patients with hazardous drinking and co-occurring mental health symptoms. This study has several strengths, including Aim 1’s collection of perspectives from key stakeholders (patients, providers, policymakers) in focus groups (and interviews) that facilitate discussion of different views, and Aim 2’s randomized controlled design. One potential limitation is the generalizability of findings given the veteran patient sample. However, VA is the largest US health care system, and according to performance data, VA-provided health care is better than or similar to that in non-VA health care systems [54]. In addition, secondarily, the current study will explore factors that explain the relationship between treatment condition and drinking and mental health symptom outcomes. Although this is a strength of the study, a limitation may be its ability to examine these mechanisms with adequate power. The present study builds on VA’s substantial efforts to ensure that primary care patients receive adequate care for hazardous alcohol use [3].

Study findings are likely to have implications for clinical practice to enhance current approaches to linking patients with HD + to alcohol care by applying a more intensive (than usual care) yet practical intervention such as C2C. The results may improve treatment outcomes for people with HD + by using patients’ strengths to problem-solve barriers to care following a process of shared decision-making with a coach. In addition to possibly accelerating the translation of C2C into practice, study findings will also support additional research in terms of a planned hybrid trial, adding to this study’s potential for high impact. Specifically, should preliminary findings suggest C2C is effective, data from Aim 1 interviews will be used to inform the selection of potential implementation strategies to be tested in a subsequent, fully powered hybrid RCT [32]. For example, Aim 1 interviews with providers and national policy leaders may identify the need for discrete implementation strategies such audit and feedback (e.g., feedback on the percentage of patients meeting screening criteria who link to alcohol care), need for educational meetings and materials, and ongoing training and identification of early adopters and champions to promote C2C’s adoption and sustained use in primary care. A subsequent trial will simultaneously examine the effectiveness of C2C and the feasibility and impact of selected implementation strategies on C2C’s adoption in VA primary care.

## Conclusion

Strengths-based approaches such as C2C can increase congruence between patients’ values and their care choices, improve provider-patient communication, and increase the likelihood that a care choice will be made [55]. C2C’s approach is recommended for optimizing care decision making for persons with substance use and mental health disorders and is effective for linking patients with comorbidities to substance use-related care [21, 22, 29]. C2C considers and emphasizes patients’ strengths such as their perseverance and dependability and helps problem-solve barriers to facilitate initiation of preferred alcohol care options. Because patients with HD + want care choices and an active role in care decision-making, interventions such as C2C that offer options and do not promote a particular care preference are likely to be more effective in helping patients initiate and engage in care and improving their outcomes.

## Data Availability

Not applicable.

## Abbreviations

AUD: Alcohol Use Disorder
C2C: Connect To Care
HD +: Hazardous Drinking and PTSD and/or Depression
PTSD: Post Traumatic Distress Disorder
HD: Hazardous Drinking
VA: Veterans Affairs
VHA: Veterans Health Administration
AR: Arkansas
USA: United States of America
CA: California; COVID: Coronavirus Disease
ADDIE: Analysis, Design, Development, Implementation, and Evaluation
CDW: Corporate Data Warehouse
PHQ: Patient Health Questionnaire
PC: Primary Care
PCL-5: Post Traumatic Stress Disorder Checklist
DSM-V: Diagnostic and Statistical Manual of Mental Disorders, 5^th^ Edition
AUDIT-C: Alcohol Use Disorders Identification Test Consumption
MOCA: Montreal Cognitive Assessment
UC: Usual Care
TLFB: Time Line Follow Back
ASSIST: Alcohol, Smoking, and Substance Involvement Screening Test
ASI: Addiction Severity Index
GEE: Generalized Estimating Equations
GLM: General Linear Models
RMSEA: Root Mean Square Error Approximation
SRMSR: Standardized Root Mean Squared Residual
CFI: Confirmatory Fit Index
US: United States
RCT: Randomized Controlled Trial
CHOICE: Choosing Healthier Drinking Options in Primary Care
SUMMIT: Substance Use Motivation and Medication Integrated Treatment
